# Effectiveness of Virtual - Compensatory Cognitive Training (V-CCT) for improving cognition in persons with schizophrenia - a multi- centre randomized controlled trial

**DOI:** 10.1186/s13063-024-08568-x

**Published:** 2024-11-01

**Authors:** Sonia Sims, Suvarna Jyothi Kantipudi, Yogitha Ashok, B. Nisha, Subhashini Gopal, Elsa Joseph, S. Amritha, Lakshmi Venkatraman, Pratiksha Venkatasubramanian, Padmavati Ramachandran

**Affiliations:** 1 Schizophrenia Research Foundation (I), Chennai, India; 2 University of California Berkeley, Berkeley, CA USA; 3 Department of Psychiatry, SRMC & RI, Sri Ramachandra Institute of Higher Education and Research (SRIHER), Chennai, India

**Keywords:** Cognition, Compensatory strategies, Compensatory cognitive training (CCT), Virtual—Compensatory Cognitive Training (V-CCT), Schizophrenia, Randomized controlled trial, Cognitive rehabilitation, Cognitive remediation

## Abstract

**Background:**

Cognitive deficits are the core component in persons with schizophrenia which impacts their socio-occupational functioning. Also, cognitive deficits cause significant impairment with the person’s quality of life [3]. Hence, targeting such a pivotal aspect in persons with schizophrenia through suitable interventions is very important. Developed countries have designed various cognitive remediation programs using computers involving high-end software which cannot be generalized to low-resource settings, like India, due to various factors including sociocultural factors, educational standards, and living standards of the patient population. Compensatory cognitive training (CCT) was developed to be “brief, practical, low-tech” and found to be effective in the west [9]. As there are no structured cognitive intervention modules in India, we have adapted the English CCT manual to be used for an urban population in Chennai, India. CCT was found to be feasible in face-to-face group sessions in our setting [12] and is found to be feasible and acceptable as virtual one–one intervention (unpublished data). Therefore, this study aims to evaluate the effectiveness of V-CCT in enhancing cognition and socio-occupational functioning.

**Methods:**

The proposed study will be a multicenter assessor-blinded randomized controlled trial at two clinical sites in Chennai, India. The preparatory phase of the study would include translation of the manual to the local language, recruitment and training of research assistants, and pilot testing using the translated manual. The second phase will be the main randomized controlled trial (RCT), during which a total of 160 persons diagnosed with schizophrenia will be recruited from both sites. After obtaining informed consent, baseline assessments will be conducted on cognition, functioning, self-esteem, and quality of life. Participants will be randomly assigned to either the virtual CCT group or the control group using a computer-generated randomization table. End-line assessments will be conducted 6 weeks after the baseline by research assistants who are blinded to the randomization post-intervention.

**Discussion:**

If V-CCT is found to be effective, it will be available for use in Tamil for persons with schizophrenia, and it will have an effect on their functioning, quality of life, and self-esteem.

**Trial registration:**

The study is registered under Clinical trial registry-India (CTRI), and the registration number is CTRI/2024/04/065267. Registered on April 2024.

## Administrative information

**Table:** 

Title {1}	Effectiveness of Virtual—Compensatory Cognitive Training (V-CCT) for improving cognition in persons with schizophrenia—A multi- center randomized controlled trial Suggest Acronym—V-CCT-CogSZ
Trial registration {2a and 2b}	The trial was registered in the Clinical Trial Registry-India (CTRI)- The registration number is CTRI/2024/04/065267
Protocol version {3}	Version 2
Funding {4}	Indian Council of Medical Research (ICMR)
Author details {5a}	Sonia Sims, Suvarna Jyothi Kantipudi*, Yogitha Ashok, Nisha Babu, Subhashini Gopal, Elsa Joseph, Amritha S, Lakshmi Venkatraman, Pratiksha Venkatasubramanian, Padmavati Ramachandran * Corresponding author
Name and contact information for the trial sponsor {5b}	There is no trial sponsor for the study
Role of sponsor {5c}	The funder has no role in study design; collection, management, analysis, and interpretation of data; writing of the report; and the decision to submit the report for publication

### Introduction

#### Background and rationale {6a}

Cognitive deficits are the core component in persons with schizophrenia, impacting their socio-occupational functioning. These deficits affect a person’s ability to lead an independent life, maintain social relationships, get employment, and sustain in them [1]. Cognitive impairments are persistent over time and do not respond to pharmacological treatment [2]. In addition to socio-occupational functioning, cognitive deficits significantly impair a person’s quality of life [3]. Hence, targeting such a pivotal aspect in persons with schizophrenia through suitable interventions is very important.

Cognitive remediation (CR) for schizophrenia, as defined by the Cognitive Remediation Experts Workshop (2010), is a behavioral training-based intervention aimed at improving cognitive processes with the goal of durability and generalization [4]. The literature on cognitive remediation therapy (CRT) demonstrates its effectiveness in improving the social and community functioning in persons with schizophrenia. Based on this evidence, the concept of functional outcome has been reconceptualized as a spectrum: a continuum from neuropsychological performance to social community functioning [5]. A meta-analysis conducted by Wykes et al. reported that cognitive remediation therapy was more effective when patients were clinically stable. Significantly stronger effects on functioning were found when cognitive remediation therapy was provided along with other psychiatric rehabilitation, and a much larger effect was present when a strategic approach was adopted with adjunctive rehabilitation [6].

Developed countries have designed various cognitive remediation programs using computers involving high-end software like NEAR (Neurocognitive and Educational Approach to (Cognitive) Remediation) and CIRCUIT (*C*omputerized *I*nteractive *R*emediation of *C*ognition and *T*hinking *S*kills). These programs utilize compensatory and restorative approaches using the learning principles [7, 8]. However, these programs cannot be generalized to low-resource settings like India due to various factors, including socio-cultural factors, educational standards, living standards of the patient population, budget restrictions, and limited mental health resources. Cognitive interventions are resource intensive, typically requiring the patients to visit the facility for 2–3 sessions per week, often accompanied by a family member. This incurs huge costs from their end, and caregivers are burdened in this process. Moreover, the shortage of mental health professionals exacerbates these challenges and must be kept in mind while developing or implementing these interventions. Considering these limitations, exploring the use of peer support volunteers or persons with lived experience, as well as virtual platforms, is crucial. Hence, it is imperative to develop culturally appropriate, feasible, low-cost, and less resource-intensive manualized cognitive interventions for use in low- and middle-income countries (LAMIC).

### Rationale of the study

Compensatory cognitive training (CCT) was developed to be “brief, practical, low-tech, engaging to clients, and portable enough to be delivered in the community” [9]. It teaches strategies to help clients work around their deficits, linking strategies to goals and roles in the community [10]. A meta-analysis on CCT identified 26 studies across 7 countries and reported a medium effect on functioning and relative durability of effects on cognition [11]. As there are no structured cognitive intervention modules in India, we have adapted the English CCT manual for use in an urban population in Chennai, India. Our study found CCT to be feasible in face-to-face group sessions in our setting [12].

Virtual psychological interventions have been tested and used before and during the pandemic [13–16]. The National Mental Health Survey 2016 also recommends the use of technology to improve services. The SCARF team has piloted virtual one-on-one CCT (V-CCT) and found it to be feasible and acceptable as a virtual intervention. This was delivered using the culturally adapted English version of CCT. According to the unpublished data, 80% of the participants completed all 12 sessions of V-CCT. Pre-post differences were observed in specific domains of cognition. The participants reported benefits such as reduced travel time and cost and made them feel independent as they did not have to rely on their caregivers to attend sessions.

### Objectives {7}

The primary objective of the study is to evaluate the effectiveness of V-CCT in enhancing cognition and socio-occupational functioning among persons with schizophrenia compared to a control intervention. The secondary objective is to assess the effectiveness of V-CCT in improving quality of life and self-esteem.

### Trial design {8}

The proposed study will be a multicenter assessor-blinded randomized controlled trial conducted at two clinical sites in Chennai, India. It will be a parallel group study, with a 1:1 allocation ratio, evaluating superiority of V-CCT over an active control intervention. The intervention sessions for both the groups will be delivered virtually, and participants will join the sessions from home using a tablet/computer.

## Methods: participants, interventions, and outcomes

### Study setting {9}

The study will be conducted in two urban sites:

1. Schizophrenia Research Foundation (SCARF), Chennai, India.
2. Sri Ramachandra Institute of Higher Education and Research (SRIHER), Chennai, India.

### Eligibility criteria {10}

Participants will be screened and selected at the study sites based on the following inclusion and exclusion criteria.

Inclusion criteria:

- ❖ Persons diagnosed with Schizophrenia as per ICD 11 classification
- ❖ Aged 18–55 years old
- ❖ Clinically stable patients with a Clinical Global Impressions (CGI) score ≤ 3, receiving outpatient treatment
- ❖ Duration of illness ≥ 2 years
- ❖ Capacity to provide informed consent
- ❖ Ability to communicate in the local language Tamil

Exclusion criteria:

- ❖ Persons reporting significant impairment in vision and/or hearing that will affect their ability to participate in the trial
- ❖ Current comorbid substance-use disorder
- ❖ Persons with organic brain conditions, intellectual disability, or physical illnesses that impede their ability to participate in the trial
- ❖ History of receiving any interventions targeting cognitive deficits in the past

### Who will take informed consent? {26a}

Researchers who have been trained in good clinical practice and the informed consent procedure will screen the eligible participants for their consent to the study. An information sheet and consent form in the local language will be provided to the participant to read. The researcher will then use a specially designed flipchart to explain the study, including the study process, the concept of randomization, risks, benefits, privacy, and confidentiality. Researchers will address any questions or concerns the participant may have. Participants will be given ample time to review the study details and discuss them with their family members before providing consent. Once the participant’s capacity to consent is ascertained and the participant is ready to consent, they will be asked to sign two consent forms, one of which will be kept by the researcher in a locked filing cabinet and the other one will be given to the participant. Any reasons for refusal to provide consent will be documented.

### Additional consent provisions for collection and use of participant data and biological specimens {26b}

In order to facilitate fidelity assessments, additional consent for audio/video recording of some sessions will be requested from the participants. The study does not include the collection of any biological samples.

## Interventions

### Explanation for the choice of comparators {6b}

We chose to include an active comparator in the control group to ensure engagement and retention in the study as well as to control for the non-specific effects of social interaction and structured activity. While usual care or a waitlist control were considered, we decided that active control with structured sessions (comprising fun games and befriending activities) would allow us to better isolate the specific effects of the V-CCT intervention by maintaining a similar structure, frequency, and duration between groups. The control activities were carefully selected to ensure no direct cognitive enhancement, thereby minimizing any confounding cognitive effects. The control intervention will be delivered by a trained facilitator.

### Intervention description {11a}

The V-CCT intervention, comprising 12 sessions, will be delivered online as a group intervention. Two sessions (1.5 to 2 h per session) per week will be delivered by a trained and certified facilitator. The 12 modules of the CCT intervention cover prospective memory, conversational and task attention, verbal learning and memory, cognitive flexibility, and problem solving.

### Criteria for discontinuing or modifying allocated interventions {11b}

No major adverse events are anticipated due to the interventions. However, in the event that a participant experiences significant psychological distress during a session, the facilitator will pause the session briefly and make sure the participant is able to continue. The participant will be given some time out of the session to recover. If necessary, and the participant continues to feel distressed, they may choose to leave the session. All the participants in the trial will remain in clinical care throughout the study period, and they will be encouraged to seek help from the clinical team.

### Strategies to improve adherence to interventions {11c}

The facilitators will inform participants about the session dates and times. Reminders will be sent to participants both 1 day and 2 h before each session.

### Relevant concomitant care permitted or prohibited during the trial {11d}

During the study period, participants will continue to receive their usual treatment, including pharmacological and psychosocial interventions. However, they will be prohibited from receiving any other cognitive interventions.

### Provisions for post-trial care {30}

The likelihood of participants encountering any harm during the study is extremely low. Should a participant disclose information suggesting an immediate risk to themselves or others, their participation will be immediately discontinued, and a researcher will promptly inform the relevant safeguarding authorities (e.g., clinic). All researchers will be well-versed in safeguarding procedures and will undergo ongoing supervision to ensure adherence to policies and protocols for handling persons who disclose potential risks. The research will be conducted exclusively by trained and proficient researchers experienced in assisting persons dealing with mental health issues. Once the study is completed, if the V-CCT intervention is found to be beneficial, it will be offered to the participants in the control arm.

### Outcomes{12}

Quantitative outcome measures will be assessed at three distinct time points: baseline (v0), post-intervention (v6) at 6 weeks, and at follow-up, which occurs 3 months from baseline (v12).

The primary outcome measures will include assessments of cognition using Brief Assessment of Cognition in Schizophrenia (BACS) [17] and Subjective Scale to Investigate Cognition in Schizophrenia (SSTICS Tamil version) [18] and functioning using Socio-Occupational Functioning Scale (SOFS) [19], at 6 weeks.

Secondary outcome measures will include assessments of quality of life using the Manchester Short Assessment of Quality of Life (MANSA) [20] and self-esteem using Rosenberg Self-Esteem Scale (RSES) [21].

All the measures will be repeated at 12 weeks to assess if the treatment effects are sustained.

The sociodemographic details of the participants will be collected at baseline and summarized as means and proportions. The primary and secondary outcome measures will be presented using mean and standard deviation, and comparisons will be made between the treatment and control arm at 6 weeks and 12 weeks. A subgroup of 20 participants, randomly selected from the intervention group, will be asked to participate in qualitative interviews at the conclusion of the 6-week intervention. These interviews aim to explore participants’ experiences with the intervention, including identifying facilitators and barriers, suggesting adaptations, and assessing the practical delivery of the intervention.

### Participant timeline {13}

**Table:** The following table explains the schedule of enrolment, intervention, and assessment

	Timeline for the study									
	Screening	Baseline	Week							Completion
Week			1	2	3	4	5	6	7	13
**Enrolment:** **Eligibility screening** **Informed consent** **Baseline assessments** **Allocation**										
	**X**									
	**X**									
		**X**								
		**X**								
**Intervention period in both arms**			**X**	**X**	**X**	**X**	**X**	**X**		
**Assessments** **Socio-demographic proforma** **BACS** **SOFS** **SSTICS** **MANSA** **RSES** **Semi structured Interview**		**X**								
		**X**							**X**	**X**
		**X**							**X**	**X**
		**X**							**X**	**X**
		**X**							**X**	**X**
		**X**							**X**	**X**
									**X**	

*BACS*, Brief Assessment of Cognition in Schizophrenia

*SOFS*, Socio-Occupational Functioning Scale

*SSTICS*, Subjective Scale to Investigate Cognition in Schizophrenia

*MANSA*, Manchester Short Assessment of Quality of Life

*RSES*, Rosenberg Self-Esteem Scale

### Sample size {14}

A Sample size of 104 participants (52 in each arm) is required based on the outcome indicators of cognitive performance with an effect size of 0.64 between the intervention and control groups, with 90% power and 5% alpha error using R 4.0.3 “pwr.t.test” software. A dropout rate of 50% has been assumed based on previous experiences and studies done using CCT. Therefore, the total number of recruited subjects at both sites will be 156 rounded up to 160 participants in total [22].

### Recruitment {15}

To ensure adequate recruitment from both the sites, posters containing study details will be displayed, inviting participants to approach the research team. Additionally, the clinicians at both the sites will be sensitized to the study criteria and encouraged to refer potential participants to the research team. In instances where patients have limited time available, the research team will divide the consent and screening process to ensure that patients are not excluded due to time constraints. To ensure fairness, participants who lack access to a laptop or tablet will receive a tablet along with an internet package to facilitate their participation in the study interventions.

## Assignment of interventions: allocation

### Sequence generation {16a}

The participants will be randomly assigned to either V-CCT or active control group in a 1:1 allocation, using a computer-generated randomization table.

### Concealment mechanism {16b}

The randomization table will be stored in password-protected computers, with access restricted to the study coordinator only.

### Implementation {16c}

Participants will be recruited by the research assistants. Once 16 consecutive participants have been recruited, randomization will be done. The allocation sequence will be created by the statistician. Subsequently, the unblinded study coordinator will notify participants of their assigned allocation group.

## Assignment of interventions: blinding

### Who will be blinded {17a}

The principal investigator (PI) is blinded to maintain objectivity in overseeing the trial, while the outcome assessors are blinded to ensure unbiased assessment of outcomes. The statistician responsible for randomization is unblinded. Both the co-investigator (Co-I) and project coordinator are unblinded, as they are conducting fidelity checks and in-depth interviews. Research assistants delivering the intervention are also unblinded. Due to the nature of the interventions, participants are not blinded and may be able to identify which intervention they are receiving.

### Procedure for unblinding if needed {17b}

If harm occurs to a participant, unblinding is permissible and will be managed in accordance with specific safeguarding policies and standard operating procedures (SOPs) established.

## Data collection and management

### Plans for assessment and collection of outcomes {18a}

All assessment tools have been translated into the local language. The study team has undergone training in good clinical practice and outcome assessment. Practice sessions for outcome assessments were conducted and overseen by SG, ensuring good inter-rater reliability. Facilitators of the intervention and active control sessions received training from Dr. Frances Dark, Metro South Health Service, Brisbane, and SG. Information will be gathered through paper case report forms (CRF) and subsequently entered into an electronic database known as REDCap. The researchers are responsible for creating and managing the REDCap database, and data cleansing will occur once the study concludes. Those interested can request access to the data collection forms from the authors.

The data assessment tools used in the study include:

- *Brief Assessment of Cognition in Schizophrenia (BACS)*: BACS includes tests of verbal memory (Verbal Memory Task), verbal working memory (Digit Sequencing Task), motor/speed (Token Motor Task), verbal fluency (Verbal Fluency Task), attention/information processing (Symbol Coding Task), and executive function (Tower of London Task). BACS scores will be converted to z-scores to provide a standard metric for combining test scores into domains and comparing performance over time, relative to healthy persons [17].
- *Socio-Occupational Functioning Scale (SOFS)*: SOFS is a brief and comprehensive scale that has been validated in India. It consists of 14 items to measure the social functioning of patients with schizophrenia across three domains, namely adaptive living skills, social appropriateness, and interpersonal skills. The items are scored on a 5-point scale, where “1” indicates no impairment, and “5” indicates extreme impairment. The ratings may be provided by mental health professionals, family members, or other care providers familiar with the patient. SOFS demonstrates good internal consistency (*α* = 0.91) and test–retest reliability, with intraclass coefficient of 0.95. It is reported to have satisfactory content validity, with several other types of validities established, including concurrent validity, criterion validity, discriminant validity, and construct validity [19].
- *Socio-demographics Questionnaire*: a questionnaire to collect socio-demographic information from all participants at baseline.
- *Subjective Scale to Investigate Cognition in Schizophrenia (SSTICS Tamil version)*: SSTICS is a self-report measure that is brief, with 21 items to assess complaints about memory, attention, praxia, and executive function in persons with schizophrenia. It demonstrates good internal consistency (*α* = 0.86) and test–retest reliability (rs = 0.82). The scale takes approximately 6 min to complete [18].

Secondary outcome measures include:

- *Manchester Short Assessment of Quality of Life (MANSA)*: The MANSA is a brief instrument for assessing quality of life focusing on satisfaction with life as a whole and with life domains. It has 16 items and is rated on a 7-point Likert scale. Cronbach’s alpha for satisfaction ratings was 0.74. Tamil version of MANSA is available and has been used for assessing subjective quality of life in persons with psychosis in the local setting [20].
- *Rosenberg Self-Esteem Scale (RSES)*: This is a self-report measure that consists of 10 items that assess the self-esteem of the respondent. The items are scored on a 4-point scale, ranging from strongly agree to strongly disagree. The test–retest reliabilities range from 0.82 to 0.88 and Cronbach’s alpha range from 0.77 to 0.88. Validity was established by its correlation with the Coopersmith Self-Esteem Inventory. Several studies have established the psychometric properties of the RSES [21].

### Plans to promote participant retention and complete follow-up {18b}

Both intervention and active control sessions will be delivered virtually for 6 weeks, consisting of two sessions per week. This schedule may potentially result in participant dropout due to various reasons. To ensure better retention rates and complete follow-up, the facilitators of both the intervention and active control groups will create an independent WhatsApp group for participants in each arm where all updates regarding the sessions will be posted.

Participants who drop out will still be invited to the end-line and follow-up assessments, unless they explicitly express disinterest. Flexible scheduling options will be provided for assessments and intervention sessions to accommodate participants’ availability based on the consensus of the group.

A subsample of participants who have dropped out of the intervention will be approached for a semi-structured interview to understand their experiences, barriers, and facilitators in attending the sessions virtually.

### Data management {19}

The paper-based case report forms (CRFs) used for data collection will be securely stored in locked cabinets at both sites. The project coordinator will oversee the verification of completed CRFs. Should any errors be identified, the CRFs will be returned to the respective assessor for necessary corrections. Following final verification, the data will be inputted into the REDCap electronic database. Co-investigators (Co-I) will then cross-reference the entered data with the source CRFs to ensure accuracy. Detailed data management protocols are outlined in the “Document Completion, Transport, and Storage SOP” and “Data Entry SOP” established by each site, which can be provided upon request from the authors.

A session log will be maintained by the facilitators of both the intervention and control arm. It will record information on participant involvement, compliance with homework (HWs), duration of the session, any challenges observed during the session, and how they were handled. Also, participant attendance will be recorded in each session, and the reasons for absence during a particular session will be documented when they attend next session, or the facilitator will contact the participant over the phone and document the reason for absence. The reasons for the same will be documented in the session log.

### Assessing fidelity of the sessions

All sessions will be recorded after obtaining consent from the participants. The Co-Is and project coordinator would randomly choose 3 out of 12 recordings and assess the fidelity using the fidelity checklist.

### Confidentiality {27}

To safeguard patient confidentiality, all patient-related information will undergo pseudonymization, with each participant assigned a unique ID number. Access to the password-protected folder containing patient personal data (such as participant name and contact information) and the corresponding ID list will be restricted to the study team. Hard copies of data, including case report forms, consent forms, patient receipts, and sociodemographic forms, will be stored in locked file cabinets at both sites. Evaluations will occur in separate rooms from both participants and researchers to enhance confidentiality. Control and intervention sessions will be conducted via a secure Zoom platform to ensure data security, with group facilitators sharing Zoom links with group members via WhatsApp. Participants will be instructed not to share the link with anyone outside the group. Transcripts will be examined on secure computers accessible only to research team members. Any participant remarks quoted in the final report will remain anonymous.

### Plans for collection, laboratory evaluation, and storage of biological specimens for genetic or molecular analysis in this trial/future use {33}

This is not applicable; this study does not collect any biological data.

## Statistical methods

### Statistical methods for primary and secondary outcomes {20a}

Statistical Package for Social Sciences (SPSS) Version 21.0 will be used to analyze the data. Descriptive statistics will be used to present sociodemographic information, continuous variables will be characterized using measures of central tendency and variability, and nominal and ordinal data will be presented as proportions (frequency). The statistical tests for inferential analysis will be chosen based on discovered distributions (parametric or non-parametric), which will be assessed using probability functions and normality tests on histograms. The effectiveness of V-CCT will be evaluated using an intention-to-treat (ITT) analysis. The mean, standard deviations, and interquartile range for each of the three time points—baseline, 6 weeks, and 12 weeks—will be computed. The significance of the variations in the means (or other parameters) of the measured outcomes will be tested by the analysis.

The primary outcomes will be the BACS scores on various domains of cognition and functioning as assessed using SOFS. Self-esteem and quality of life are the secondary outcome measures. The effect size and 95% CI will be calculated to establish the superiority of the intervention arm over the control arm. Additionally, qualitative data obtained from the semi structured interview will be coded and analyzed.

### Interim analyses {21b}

No interim analysis will be conducted due to the short duration of intervention and the small sample size.

### Methods for additional analyses (e.g., subgroup analyses) {20b}

A subgroup analysis will be considered based on age and duration of illness, particularly if data suggests presence of effect modifiers.

### Methods in analysis to handle protocol non-adherence and any statistical methods to handle missing data {20c}

We will employ intention-to-treat (ITT) analysis to address protocol non-adherence, which helps preserve the randomization. To deal with any missing data points, we will employ multiple imputation techniques, which help with the retention of statistical power. We will also perform sensitivity analysis to assess the robustness our results hold up against alternative assumptions on missing data.

### Plans to give access to the full protocol, participant-level data, and statistical code {31c}

At present, full open access is not available, but we may consider granting access to the complete protocol, including participant-level data and statistical analysis, on a case-by-case basis.

## Oversight and monitoring

### Composition of the coordinating center and trial steering committee {5d}

The PI and project coordinator at SCARF, the trial’s primary coordinating center, offer the partner site programmatic, financial, and technical support. Additionally, they provide everyday guidance, support and training for the trail’s execution at each site. The trial steering committee, represented by the funder, oversees the study’s progress and provides strategic direction. The research team submits annual reports to the steering committee. The committee will contact the researchers if any clarifications or further information are required. Additionally, there is no formal patient and public involvement in this study.

### Composition of the data monitoring committee, its role and reporting structure {21a}

The ethics committee has determined that a data monitoring committee is not required for this trial, as it does not involve the use of medication.

### Adverse event reporting and harms {22}

No major adverse events are anticipated due to the interventions. However, if a participant experiences significant psychological distress during a session, the facilitator will briefly pause the session to ensure the participant can continue. The participant will be given some time to recover, and if they continue to feel distressed, they will have the option to leave the session. All participants in the trial will remain under clinical care throughout the study period and will be encouraged to seek assistance from the clinical team. While no major adverse events have been reported in the literature, one study noted that a participant dropped out due to difficulties in keeping up with the group’s pace [12]. Every adverse event (AE) and serious adverse event (SAE) will be documented in both the participant’s clinical record and the primary research file. The project coordinator will receive the report and forward it to the principal investigator. Every adverse event will be assessed by the PI, who will also assess its severity and take appropriate action. The ethics committee will be notified of both AEs and SAEs.

### Frequency and plans for auditing trial conduct {23}

Monitoring audit and inspection will be carried out by the funding agency as and the Institutional Ethics Committee from both study sites, as and when needed.

### Plans for communicating important protocol amendments to relevant parties (e.g., trial participants, ethical committees) {25}

Any changes made after the trial has started will be reported for approval to the ICMR and SCARF ethics committee, and the version number and date of each update will be used to monitor the history of those changes.

### Dissemination plans {31a}

A comprehensive study report will be provided to the funding agency, and the findings will be submitted for publication in peer-reviewed journals.

Once the research study is completed, the results will be shared in several ways to reach different groups of people. Our main goal is to ensure that the findings benefit both the scientific community and the public.

1. *For participants and the public*: We will prepare easy-to-understand summaries of the results, which will be shared with all study participants and made available to the public through newsletters, websites, and social media platforms. These summaries will explain what we learned from the study, how it might help people with schizophrenia, and what it means for cognitive training in the future
2. *For healthcare providers*: The findings will be shared with doctors, mental health professionals, and caregivers through workshops, seminars, and conferences. This will help them understand the effectiveness of the virtual cognitive training program and how they can use it in their practice to support people with cognitive difficulties
3. *For the scientific community*: We will publish our results in scientific journals so that researchers around the world can learn from our study and build on our work. The findings will also be presented at national and international conferences to spread awareness about the virtual cognitive training approach.
4. *For policy makers and funders*: We will share the results with policy makers and health organizations to encourage the inclusion of cognitive training interventions like V-CCT in mental health programs. We will also inform our funders, the Indian Council of Medical Research (ICMR), about the outcomes of the study

By sharing our findings widely, we hope to influence future research, improve mental health care, and ultimately enhance the lives of people with schizophrenia.

## Discussion

This study aims to evaluate the effectiveness of compensatory cognitive training (CCT) in improving cognitive deficits among persons with schizophrenia, compared to an active control intervention. The CCT Intervention will be delivered through virtual platforms in a group format. The study also employs an active control intervention, manualized and translated into Tamil, comprising various fun and befriending activities. The active control intervention helps control for the experience of a group intervention online from home with a facilitator. In addition to assessing improvements in cognition and functioning, the study intends to assess if cognitive interventions can bring a change in quality of life and self-esteem. One of the key strengths of this study is its randomized controlled trial (RCT) design, which effectively controls for baseline confounding factors. Furthermore, it utilizes a superiority study design to test whether the V-CCT intervention out forms the active control arm in improving cognition. While the use of manualized interventions enhances standardization and replicability, there are several limitations to consider. These include digital literacy challenges, and a higher risk of missing data, given the population of persons with lived experience of schizophrenia. In order to address these concerns, we plan to recruit 160 participants, assuming the 50% drop out rate.

We will employ a comprehensive approach to enhance reliability and validity of our study, by including ITT for protocol non-adherence, multiple imputation for missing data, and sensitivity analysis to check robustness of our findings.

## Trial status

Protocol version 2, 23.04.2024. The preliminary phase of the study was initiated on 1 December 2023. Translation of the intervention manual to the local language and development of active control manual have been completed, and the pilot phase has been initiated. The main RCT phase is scheduled to commence by June 15, 2024, with recruitment anticipated to conclude by December 31, 2025.

## Data Availability

The final trial dataset from both the study sites will be held on the REDCap server, which will be managed by the IT department at SCARF. All the researchers from the study team will have access to the final trial dataset. Any data required to support the protocol can be supplied on request. The data supporting the findings of this study are available upon reasonable request, subject to approval from the funding agency.

## Abbreviations

V-CCT: Virtual-Compensatory Cognitive Training
CCT: Compensatory cognitive training
RCT: Randomized controlled trial
CTRI: Clinical Trial Registry-India
ICMR: Indian Council of Medical Research
CR: Cognitive remediation
CRT: Cognitive remediation training
NEAR: Neurocognitive and Educational Approach to (Cognitive) Remediation
CIRCUIT: Computerized Interactive Remediation of Cognition and Thinking Skills
LAMIC: Low- and middle-income countries
CRF: Case report forms
REDCap: Research Electronic Data Capture
SOP: Standard operating procedure
SCARF: Schizophrenia Research Foundation
SRIHER: Sri Ramachandra Institute of Higher Education and Research
ICD: International Classification of Disorders
CGI: Clinical Global Impression
GCP: Good Clinical Practice
SPSS: Statistical Package for Social Sciences
ITT: Intent to treat
BACS: Brief Assessment of Cognition in Schizophrenia
SSTICS: Subjective Scale to Investigate Cognition in Schizophrenia
MANSA: Manchester Assessment Scale for Quality of Life
SOFS: Social Occupational Functioning Scale
RSES: Rosenberg Self-Esteem Scale
PI: Principal investigator
CO-I: Co-investigator
HW: Homework
